# Treatment of Urolithiasis with Medicinal Plant* Salvia miltiorrhiza*: A Nationwide Cohort Study

**DOI:** 10.1155/2018/8403648

**Published:** 2018-04-11

**Authors:** Wen-Chi Chen, San-Yuan Wu, Po-Chi Liao, Tzu-Yang Chou, Huey-Yi Chen, Jen-Huai Chiang, Yuan-Chih Su, Kee-Ming Man, Ming-Yen Tsai, Yung-Hsiang Chen

**Affiliations:** ^1^Graduate Institute of Integrated Medicine, Chinese Medicine Research Center, Research Center for Chinese Medicine & Acupuncture, College of Medicine, China Medical University, Taichung, Taiwan; ^2^Departments of Urology, Obstetrics and Gynecology, Medical Research, and Anesthesiology, Management Office for Health Data, China Medical University Hospital, Taichung, Taiwan; ^3^Center for General Education, Feng Chia University, Taichung, Taiwan; ^4^Center for General Education, Chaoyang University of Technology, Taichung, Taiwan; ^5^Department of Urology, Taichung Veterans General Hospital, Taichung, Taiwan; ^6^Department of Chinese Medicine, Kaohsiung Municipal Gangshan Hospital, Kaohsiung, Taiwan; ^7^Department of Medicinal Botanicals and Health Applications, Da-Yeh University, Changhua, Taiwan; ^8^Department of Chinese Medicine, Kaohsiung Chang Gung Memorial Hospital and Chang Gung University College of Medicine, Kaohsiung, Taiwan; ^9^Department of Psychology, College of Medical and Health Science, Asia University, Taichung, Taiwan

## Abstract

*Salvia miltiorrhiza* Bunge (Danshen), a common medicinal plant in traditional Chinese medicine, has been tested effectively to prevent urolithiasis in animals; nevertheless, the clinical application for urolithiasis remains unclear. We thus investigated the clinical effect of Danshen by analyzing the database from the Taiwan National Institute of Health. The cohort “Danshen-users” was prescribed Chinese herb medicine Danshen after the initial diagnosis of calculus. The control group (non-Danshen-users) was not given Danshen after the initial diagnosis of calculus. The date of first using Danshen after new diagnosis date of calculus was considered as index date. The outcome variables were categorized into two categories: the first category included calculus surgical treatment, including extracorporeal shock wave lithotripsy, ureteroscopy, percutaneous nephrostomy with fragmentation, and ureterolithotomy; the second category included any bleeding disorders, including gastrointestinal bleeding, intracranial hemorrhage, and blood transfusions. The incidence of calculus surgical treatment in the Danshen-users was less than that in the non-Danshen-users: 1.071% in 1,000 person-years (200 people followed up for 5 years) and 3.142% in 1,000 person-years, respectively. The adjusted hazard ratio for calculus surgical treatment in the Danshen-users was 0.34 (95% confidence intervals: 0.31–0.38) as compared to the non-Danshen-users. When stratified by sex, the incidence of calculus surgical treatment in Danshen-users was 0.685% in 1,000 person-years and 1.575% in 1,000 person-years for women and men, respectively, which was lower than that in non-Danshen-users. Danshen decreased the ratio of subsequent stone treatment after the first treatment in the study population; there was no increased bleeding risk due to long-term Danshen use.

## 1. Introduction

Urolithiasis is a common urological disorder with an annual incidence of 7–13% in North America, 1–5% in Asia [1, 2], and 6.4% in Taiwan [3]. Urolithiasis is also a disease with high recurrence. Over 50% of the patients with stone experience stone episode recurrence after 5 years of their first treatment [4]. Therefore, seeking drugs for the prevention of stone recurrence is an important issue.

*Salvia miltiorrhiza* Bunge (Danshen) is a common medicinal plant in traditional Chinese medicine (TCM) with its roots (dried) possessing pharmacological properties [5]. Danshen is a classical Huoxue Huayu herb (a TCM term used for activating blood circulation, relieving pain, activating blood to promote menstruation, clearing heart fire, tranquilizing, and treating blood stasis) that has been prescribed clinically for one thousand years [6]. In modern medicine, Danshen is used for the treatment of cardiovascular diseases [7, 8], osteoporosis [9], anticancer [5], and hepatoprotective effect [9]. Danshen is one of the tested effective TCM herbs for prevention of stone disease in our previous study [10, 11]. We chose Danshen (as an herb to be tested) because of its effectiveness in the treatment of blood disorders. According to TCM, blood stasis is one of the major pathogeneses of stone disease, and hematuria is frequently observed in patients with stones.

We used the database from the National Institutes of Health (NIH), Taiwan, to study the clinical effect of the potential TCM herb on urolithiasis. Although Danshen has been tested effectively for the prevention of urolithiasis in animal models [11], the clinical application in the prevention of urolithiasis is still under investigation [12]. The objective of the present study was to investigate the preventive effect of Danshen clinically by analyzing the NIH database. The surrogate outcome will be a decrease in the number of stone surgeries in a cohort. We have also studied the possible effects of increased bleeding tendency due to the antiplatelet effect of Danshen used for treating blood stasis.

## 2. Materials and Methods

### 2.1. Database

For this retrospective cohort study, our data source was from National Health Insurance Research Database (NHIRD) in Taiwan. Taiwan's National Health Insurance (NHI) program is a compulsory insurance that has been providing comprehensive coverage to 99% of 23 million individuals since 1996. The NHIRD included information of sex, birthday, outpatient care, inpatients care, western and traditional Chinese medicine (TCM) prescription, medical institutions, and registration files with scrambled identifications. We used the LHID 2000 (Longitudinal Health Insurance Database 2000), which contains medicine information between 1996 and 2013 of 1 million beneficiaries randomly sampled from the registry of all beneficiaries in 2000. The sampled patients exhibit no significant difference in age, sex, birth year, or average insured payroll-related amount from the general population. The International Classification of Diseases, Ninth Revision, Clinical Modification (ICD-9-CM) codes were used for diagnoses. Because the NHIRD contains identified secondary data for research, the present study was waived from informed consent. A disease diagnosis without valid supporting clinical findings may be considered a medical fraud by NHI with a penalty of 100 times of the payment claimed by the treating physician or hospital. This study was approved by the Institutional Review Board of China Medical University (CMUH104-REC2-115).

### 2.2. Study Population

All cases diagnosed with calculus (ICD-9-CM: 592.0, 592.1, and 592.9) from January 2000 to December 2010 and aged ≥18 years were the study cohort population. The case cohort population was defined as patients who were orally given (either single or formula form) herbal medicine powder Danshen after initial diagnosis of calculus. Patients did not use Danshen after initial diagnosis date of calculus as compared to cohort group. The date of first using Danshen after new diagnosis date of calculus was considered as index date.

### 2.3. Covariate Assessment

Sociodemographic factors included age and sex. Age was divided into 3 groups: 18–39 years old, 40–64 years old, and ≥65 years old. Baseline comorbidities were considered if ICD-9-CM codes appeared at least once in outpatients or inpatients before initial fibromyalgia diagnosis, including diabetes mellitus (ICD-9-CM: 250), hypertension (ICD-9-CM: 401–405), urinary tract infection (ICD-9-CM: 599.0), chronic kidney disease (ICD-9-CM: 585), and gout (ICD-9-CM: 274.9).

### 2.4. Primary Outcome

The outcome variables were two: one was calculus surgical treatment, including extracorporeal shock wave lithotripsy (ESWL), ureteroscopy, percutaneous nephrostomy with fragmentation (PCNL), and ureterolithotomy, and the other was any bleeding disorders, including gastrointestinal bleeding (ICD-9-CM: 578.0, 578.1, 578.9), intracranial hemorrhage (ICH, ICD-9-CM: 432.0, 432.9), and blood transfusions (OP code: 99.0). Each individual was assessed from the index date to 31 December 2013 (end of the study) until the time of diagnosis of calculus surgical treatment or any bleeding disorders or until the patients were censored for withdrawal from insurance or lost to follow-up (which one first occurs).

### 2.5. Statistical Analyses

Student's *t*-tests for continuous variables and Chi-square test for categorical variables were used to compare the two study groups. We estimated hazard ratios (HRs) and 95% confidence intervals (CI) of calculus surgical treatment and bleeding disorder for the cohort using Danshen compared to the cohort not using Danshen by Cox proportional hazard model. Statistical analysis was performed and figures were created using SAS 9.4 (SAS Institute, Cary, NC) and R software. *P* < 0.05 in two-tailed tests indicated statistical significance.

## 3. Results

Our study included a total of 8,568 patients using Danshen (Danshen-users) and 56,502 patients not using Danshen (non-Danshen-users) suffering from calculus disease [after frequency matching (1 : 1) through sex, age (per 5 years), initial diagnosis year of calculus, and index year]. There were 8,536 Danshen-users and non-Danshen-users in each cohort.  Table 1 shows the characteristics of both groups. The mean age (standard deviation, SD) for Danshen-users and non-Danshen-users was 46.40 (14.29) years and 46.42 (14.30) years, respectively. After frequency matching, the distribution of sex and age was not significantly different (*P* = 0.99 for both) between Danshen-users and non-Danshen-users. The proportion of baseline comorbidities in Danshen-users was higher than that in non-Danshen-users (*P* < 0.05 for all). The mean (median) follow-up period for Danshen-users and non-Danshen-users was 6.27 (5.98) years and 5.09 (4.86) years, respectively.

The incidence of calculus surgical treatment in the Danshen-users was less than that in the non-Danshen-users, 1.071% in 1,000 person-years (200 people followed up for 5 years) and 3.142% in 1,000 person-years, respectively. The adjusted hazard ratio (HR) for calculus surgical treatment in the Danshen-users was 0.34 (95% CI: 0.31–0.38) as compared to the non-Danshen-users. When stratifying by sex, the incidence of calculus surgical treatment in the Danshen-users was 0.685% in 1,000 person-years and 1.575% in 1,000 person-years for women and men, respectively, which was lower than that in the non-Danshen-users (2.454% in 1,000 person-years and 4.070% in 1,000 person-years for women and men, resp.).

**Figure 1:**
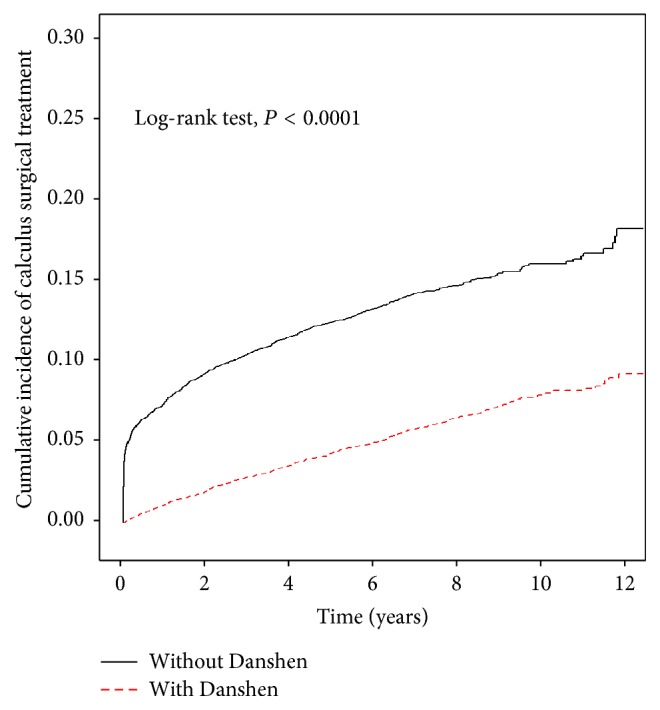
The estimated cumulative incidence of calculus surgical treatment in the Danshen-users was lower than that in the non-Danshen-users by Kaplan-Meier analysis.

When stratifying by age, the incidence of calculus surgical treatment in Danshen-users was 1.087% in 1,000 person-years, 1.137% in 1000 person-years, and 0.690% in 1,000 person-years for 18–39 years age group, 40–64 years age group, and above 65 years age group, respectively, which was lower than that in non-Danshen-users (2.675% in 1,000 person-years, 3.514% in 1,000 person-years, and 2.989% in 1,000 person-years for 18–39 years age group, 40–64 years age group, and above 65 years age group, resp.). The adjusted HRs revealed a significantly lower risk of calculus surgical treatment in the Danshen-users as compared to the non-Danshen-users in all the categories: women, men, age group of 18–39 years, age group of 40–64 years, and age group above 65 years population (Table 2). The estimated cumulative incidence of calculus surgical treatment in the Danshen-users was lower than that in the non-Danshen-users (*P* < 0.0001, log-rank test) (Figure 1).

The incidence of any bleeding disorder in the Danshen-users was less than that in the non-Danshen-users (1.708% in 1000 person-years and 2.577% in 1,000 person-years, resp.). The adjusted HR for calculus surgical treatment in Danshen-users was 0.61 (95% CI: 0.56–0.67) as compared to the non-Danshen-users. When stratifying by sex, the incidence of any bleeding disorder in the Danshen-users was 1.524% in 1,000 person-years and 1.949% in 1,000 person-years for women and men, respectively, which was lower than that in the non-Danshen-users (2.557% in person-years and 3.007% in 1,000 person-years for women and men, resp.).

**Figure 2:**
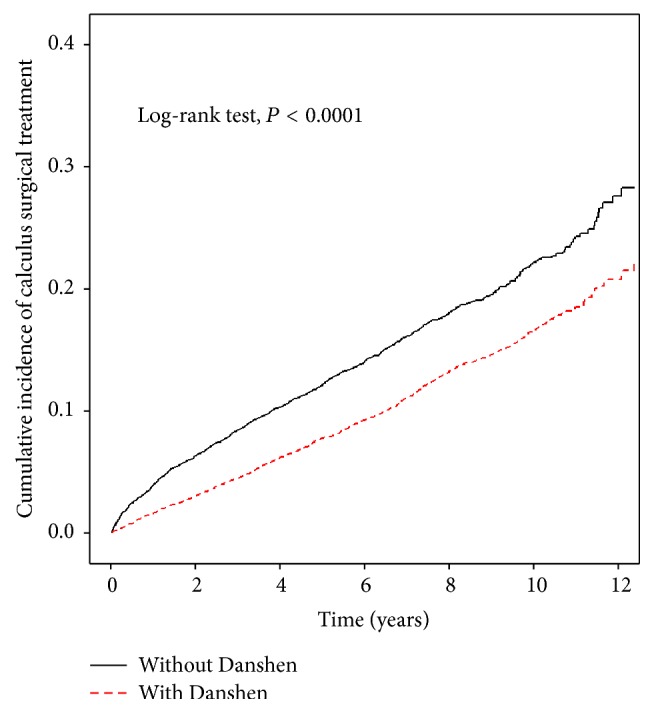
The estimated cumulative incidence of any bleeding disorder in the Danshen-users was lower than that in the non-Danshen-users by Kaplan-Meier analysis.

When stratifying by age, the incidence of calculus surgical treatment in Danshen-users was 0.819% in 1,000 person-years, 1.827% in 1,000 person-years, and 4.228% in 1,000 person-years for 18–39 years age group, 40–64 years age group, and above 65 years age group, respectively, which was lower than that in the non-Danshen-users (1.198% in 1,000 person-years, 2.723% in 1,000 person-years, and 7.307% in 1,000 person-years for 18–39 years age group, 40–64 years age group, and above 65 years group, resp.). The adjusted HRs revealed a significantly lower risk of any bleeding disorder in the Danshen-users as compared to the non-Danshen-users in all the categories: females, males, age group of 18–39 years, age group of 40–64 years, and above 65 years age group population (Table 2). The estimated cumulative incidence of any bleeding disorder in the Danshen-users was lower than that in the non-Danshen-users (*P* < 0.0001, log-rank test) (Figure 2). The HRs and 95% CI of calculus surgical treatment and any bleeding disorder associated with cumulative dose per year of Danshen among calculus patients with Danshen-users were shown in Table 3.

## 4. Discussion

We observed that Danshen significantly reduces the subsequent surgical treatment after the first stone episode, with a hazard ratio of 0.34. This effect was consistent in both sexes and among all age groups. Danshen may prove to be clinically effective for those having stone disease and seeking a measure to prevent their further surgical treatment. Danshen poses a concern regarding the increased bleeding tendency due to its enhanced blood stasis effect. However, we did not find any incidence involving hemorrhage or any transfusion event in this cohort. This result suggests that long-term use of Danshen may prove to be safe without any bleeding disorder.

The idea of using Danshen in the present study originated from our previous data [11]. Danshen revealed its preventive results for the crystal formation in a fruit fly (as observed in an animal study).* Salvia miltiorrhiza* appeared in the classic herbal book named “Shennong Ben Cao Jing” more than 2000 years ago (about 200 and 250 AD) [13]. It was described as a noble and nontoxic herb. It is often used to treat cardiovascular diseases [14, 15], hypertension, and ischemic stroke due to its efficacy on blood circulation [16, 17]. Till date, more than thirty pharmaceutical preparations for the treatment of cardiovascular diseases have been developed in clinical practice [18]. Cardiac and renal dysfunctions often occur simultaneously due to the shared causes and pathogenesis [19]. Furthermore, oxidative stress is considered as the most important determinant of the common cause [20].

The hypertensive patients have a greater risk of kidney stones than those with normal blood pressure. The patients with kidney stones are more likely to suffer from hypertension than those without urolithiasis. Thus, there exists a positive correlation between hypertension and renal stones [21]. According to a recent study, Danshen is the most frequently prescribed single herb drug for hypertension [22]. In addition, previous animal studies revealed that overproduction of reactive oxygen species causes kidney damage, and* Salvia miltiorrhiza* helps to improve the renal function and prevent oxidative stress in the renal tissues, thereby treating the renal damage [23–26].

Our previous study conducted with an emerging translational model to screen antilithic herbal drugs revealed the inhibitory effect of Danshen on the formation of CaO*x* crystals in the Malpighian tubules of* Drosophila *[11]. According to the epidemiological studies, urolithiasis is associated with many chronic diseases including obesity, metabolic syndrome, diabetes, hypertension, chronic kidney disease, and coronary artery disease [27–32]. The correlation between these chronic diseases and renal stones is most likely the result of a common pathophysiological mechanism. Reactive oxygen species (ROS) and oxidative stress are the common features between kidney stones and venereal diseases [33]. Further evidences showed that ROS is also produced in idiopathic CaO*x* kidney stones. A kidney stone is not only a physical-chemical event but also a metabolic disorder. The chronic diseases associated with oxidative stress are related to each other. Oxidative stress is usually caused by one disorder, which in turn contributes to the development of another disorder, particularly hypertension and diabetes. Both these effects may lead to oxidative stress, kidney damage, and inflammation, along with the changes in the urinary environment which promote crystallization [21]. Therefore, the treatment of urinary tract stones should not be overlooked, and the original source must be cured completely. Furthermore, TCM focuses on the reconstruction of the homeostasis prior to the formation of stones, along with acting as a treatment of urolithiasis after kidney disease and stone formation [34]. Therefore, Danshen may play an important role in the prevention of urolithiasis.

The limitations of this study include limited patient number, a surrogate outcome instead of recurrence, and unknown stone site and number. In addition, calculus surgical treatment option depends on stone size. However, LHID 2000 does not provide the information of stone size.

## 5. Conclusions

Danshen decreased the ratio of subsequent stone treatment after the first treatment in a population study from Taiwan's database. Long-term use of Danshen may prove to be safe with a reduced risk of a bleeding event. Therefore, Danshen is a safe herb having a potential for the prevention of calculus.

## Figures and Tables

**Table 1:** Characteristics of calculus patients according to use or no use of Danshen.

Variable	Using Danshen in calculus patients				*P* value^*∗*^
	No (*n* = 8536)		Yes (*n* = 8536)		
	*n*	%	*n*	%	
*Sex*					0.99
Female	4723	55.33	4723	55.33	
Male	3813	44.67	3813	44.67	
*Age group, years*					0.99
18–39	3018	35.36	3018	35.36	
40–64	4508	52.81	4508	52.81	
≥65	1010	11.83	1010	11.83	
Mean ± SD (years)	46.42 (14.30)		46.40 (14.29)		0.9157^a^
*Baseline comorbidities*					
Diabetes mellitus	2992	35.05	3339	39.12	<0.0001
Urinary tract infection	4897	57.37	5241	61.4	<0.0001
Hypertension	4402	51.57	4561	53.43	0.0148
Chronic kidney disease	657	7.7	753	8.82	0.0076
Gout	1608	18.84	1811	21.22	0.0001
*Duration between diagnosis date of calculus and index, days (mean, median)*	1120 (923)		1130 (921)		0.4553^a^

^*∗*^Chi-square test; ^a^*t*-test. The mean (median) of follow-up period was 6.27 (5.98) years and 5.09 (4.86) years for cohort group using Danshen and cohort group not using Danshen, separately.

**Table 2:** Incidence rates, hazard ratio, and confidence intervals of calculus surgical treatment and any bleeding disorder for calculus patients using and not using Danshen in the stratification of sex and age.

Variables	Using Danshen in calculus patients						Crude HR	Adjusted HR^†^
	No			Yes				
	(*n* = 8536)			(*n* = 8536)				
	Event	Person-years	IR	Event	Person-years	IR	(95% CI)	(95% CI)
*Outcome: calculus surgical treatment*								
*Total*	1370	43605	31.42	574	53589	10.71	0.36 (0.33–0.4)^*∗∗∗*^	0.34 (0.31–0.38)^*∗∗∗*^
*Sex*								
Female	615	25057	24.54	208	30354	6.85	0.3 (0.26–0.35)^*∗∗∗*^	0.29 (0.24–0.34)^*∗∗∗*^
Male	755	18548	40.70	366	23235	15.75	0.41 (0.36–0.47)^*∗∗∗*^	0.39 (0.34–0.44)^*∗∗∗*^
*Age group, years*								
18–39	449	16784	26.75	215	19782	10.87	0.43 (0.36–0.5)^*∗∗∗*^	0.4 (0.34–0.47)^*∗∗∗*^
40–64	799	22739	35.14	320	28151	11.37	0.35 (0.3–0.39)^*∗∗∗*^	0.33 (0.29–0.38)^*∗∗∗*^
≥65	122	4082	29.89	39	5656	6.90	0.26 (0.18–0.37)^*∗∗∗*^	0.25 (0.17–0.35)^*∗∗∗*^
*Outcome: any bleeding disorders*								
*Total*	1138	44166	25.77	917	53678	17.08	0.66 (0.61–0.72)^*∗∗∗*^	0.61 (0.56–0.67)^*∗∗∗*^
*Sex*								
Female	572	25341	22.57	463	30379	15.24	0.68 (0.6–0.77)^*∗∗∗*^	0.63 (0.56–0.71)^*∗∗∗*^
Male	566	18824	30.07	454	23299	19.49	0.64 (0.57–0.73)^*∗∗∗*^	0.59 (0.52–0.67)^*∗∗∗*^
*Age group, years*								
18–39	202	16862	11.98	162	19786	8.19	0.68 (0.56–0.84)^*∗∗∗*^	0.65 (0.53–0.8)^*∗∗∗*^
40–64	629	23102	27.23	516	28238	18.27	0.67 (0.59–0.75)^*∗∗∗*^	0.63 (0.56–0.71)^*∗∗∗*^
≥65	307	4202	73.07	239	5653	42.28	0.58 (0.49–0.69)^*∗∗∗*^	0.55 (0.46–0.65)^*∗∗∗*^

IR, incidence rates per 1,000 person-years; HR, hazard ratio; CI, confidence interval. Adjusted HR^†^ represented adjusted hazard ratio: mutually adjusted for Danshen drug used, sex, age, diabetes mellitus, urinary tract infection, hypertension, chronic kidney disease, and gout in Cox proportional hazard regression. ^*∗∗∗*^*P* < 0.001.

**Table 3:** Hazard ratios and 95% confidence intervals of calculus surgical treatment and any bleeding disorder associated with cumulative dose per year of Danshen among calculus patients using Danshen.

Used Danshen dose (g) per year	*n*	Number of events	Hazard ratio (95% CI)	
			Crude	Adjusted^†^
*Event: calculus surgical treatment*				
Less than 417 g per year	2813	203	1 (reference)	1 (reference)
417–1096 g per year	3025	179	0.75 (0.62–0.92)^*∗∗*^	0.76 (0.62–0.93)^*∗∗*^
More than 1096 g per year	2698	192	0.92 (0.75–1.12)	0.94 (0.77–1.14)
*Event: any bleeding disorders*				
Less than 417 g per year	2813	306	1 (reference)	1 (reference)
417–1096 g per year	3025	308	0.84 (0.71–0.98)^*∗*^	0.83 (0.71–0.97)^*∗*^
More than 1096 g per year	2698	303	0.94 (0.80–1.10)	0.95 (0.81–1.11)

Adjusted HR^†^ represented adjusted hazard ratio: mutually adjusted for sex, age, diabetes mellitus, urinary tract infection, hypertension, chronic kidney disease, and gout in Cox proportional hazard regression; ^*∗*^*P* < 0.05; ^*∗∗*^*P* < 0.01; tertiles are two cut points that will divide a dataset into three equal-sized groups. 417 g was 33rd percentage point and 1096 g was 66th percentage.
